# Reduced cardiomyocyte Na^+^ current in the age‐dependent murine *Pgc‐1β* ^*−/−*^ model of ventricular arrhythmia

**DOI:** 10.1002/jcp.27183

**Published:** 2018-08-26

**Authors:** Shiraz Ahmad, Haseeb Valli, Robert Smyth, Anita Y. Jiang, Kamalan Jeevaratnam, Hugh R. Matthews, Christopher L.‐H. Huang

**Affiliations:** ^1^ Physiological Laboratory University of Cambridge Cambridge United Kingdom; ^2^ Department of Veterinary Pre‐clinical Sciences, Faculty of Health and Medical Sciences University of Surrey Guildford United Kingdom; ^3^ Department of Physiology, PU‐RCSI School of Medicine, Perdana University Serdang Malaysia; ^4^ Department of Biochemistry University of Cambridge Cambridge United Kingdom

**Keywords:** age‐dependent arrhythmia, energetic deficiency, K^+^ currents, murine hearts, Na^+^ currents, Pgc‐1β, ventricles

## Abstract

Peroxisome proliferator‐activated receptor‐γ coactivator‐1 deficient (*Pgc‐1β* ^*−/−*^) murine hearts model the increased, age‐dependent, ventricular arrhythmic risks attributed to clinical conditions associated with mitochondrial energetic dysfunction. These were accompanied by compromised action potential (AP) upstroke rates and impaired conduction velocities potentially producing arrhythmic substrate. We tested a hypothesis implicating compromised Na^+^ current in these electrophysiological phenotypes by applying loose patch‐clamp techniques in intact young and aged, wild‐type (WT) and *Pgc‐1β* ^−/−^, ventricular cardiomyocyte preparations for the first time. This allowed conservation of their in vivo extracellular and intracellular conditions. Depolarising steps elicited typical voltage‐dependent activating and inactivating inward Na^+^ currents with peak amplitudes increasing or decreasing with their respective activating or preceding inactivating voltage steps. Two‐way analysis of variance associated *Pgc‐1β* ^*−/−*^ genotype with independent reductions in maximum peak ventricular Na^+^ currents from −36.63 ± 2.14 (*n* = 20) and −35.43 ± 1.96 (*n* = 18; young and aged WT, respectively), to −29.06 ± 1.65 (*n* = 23) and −27.93 ± 1.63 (*n* = 20; young and aged *Pgc‐1β* ^−/−^, respectively) pA/μm^2^ (*p* < 0.0001), without independent effects of, or interactions with age. Voltages at half‐maximal current V*, and steepness factors *k* in plots of voltage dependences of both Na^+^ current activation and inactivation, and time constants for its postrepolarisation recovery from inactivation, remained indistinguishable through all experimental groups. So were the activation and rectification properties of delayed outward (K^+^) currents, demonstrated from tail currents reflecting current recoveries from respective varying or constant voltage steps. These current–voltage properties directly implicate decreases specifically in maximum available Na^+^ current with unchanged voltage dependences and unaltered K^+^ current properties, in proarrhythmic reductions in AP conduction velocity in *Pgc‐1β^−/−^* ventricles.

## 1 INTRODUCTION

Cardiovascular disease is the leading worldwide clinical cause of mortality. Around half of such cases likely arise from sudden cardiac death (SCD) following ventricular arrhythmias (Go et al., 2013). Increasing evidence links the incidence of such events to both ageing and age‐related conditions compromising cardiomyocyte metabolic energetics and mitochondrial function. Thus ageing is associated with an increased incidence of cardiac rhythm disturbances including ventricular tachy‐arrhythmias (Bradshaw, Stobie, Knuiman, Briffa, & Hobbs, 2014; Deo & Albert, 2012; Go et al., 2001). Cardiac failure, diabetes, and obesity similarly constitute age‐dependent risk factors for SCD in their own right independent of any accompanying coronary artery disease (Hookana et al., 2011; Kucharska‐Newton et al., 2010; Yeung et al., 2012). However, the mechanisms for arrhythmic events in situations involving energetic abnormalities remain unclear.

The biochemical consequences of compromised cellular energetics have been studied in murine models deficient in peroxisome proliferator activated receptor‐γ coactivator‐1 (PGC‐1) transcriptional coactivators. Cardiac, brain, and skeletal muscle tissue, characterised by high oxidative activity, strongly express the key regulators of mitochondrial function, PGC‐1α and PGC‐1β (Finck & Kelly, 2006; Lin, Handschin, & Spiegelman, 2005; Riehle & Abel, 2012). These promote expression of nuclear and/or mitochondrial encoded proteins involved in the tricarboxylic acid cycle, β–oxidation, and oxidative phosphorylation (Arany et al., 2005). Their expression levels fall along with mitochondrial dysfunction in metabolic conditions including obesity, insulin resistance, type II diabetes mellitus, and ageing (Dillon, Rebelo, & Moraes, 2012; Leone & Kelly, 2011; Mootha et al., 2003).

Cardiac arrhythmias fundamentally result from disruptions in the normally coordinated sequence of ion channel activation and inactivation underlying cardiac AP excitation and its propagation. Previous studies have therefore examined the roles of individual channels in such events, often employing models with monogenic modifications or pharmacological manipulations directed at specific channels. Studies in Nav1.5‐haplo‐insufficient murine *Scn5a* ^*+/−*^ hearts have implicated loss of Na^+^ channel function and accelerated age‐related fibrotic changes in compromising AP conduction velocities. This accentuates tissue‐level re‐entrant circuit formation, ultimately resulting in the generation of the ventricular arrhythmic substrate present in the Brugada Syndrome (Huang, 2017; Jeevaratnam et al., 2011, 2012; Martin et al., 2012).

Electrophysiological studies have similarly reported ventricular arrhythmic phenotypes in *Pgc‐1β* ^−/−^ hearts (Gurung et al., 2011; Lelliott et al., 2006) including an increased incidence of alternans phenomena attributable to the effects of both advanced age and genotype in isolated Langendorff‐perfused preparations (Ahmad et al., 2017). Intact *Pgc‐1β* ^−/−^ animals also showed evidence for age‐dependent AP conduction slowing during noninvasive electrocardiographic recording (Ahmad, Valli, Salvage et al., 2018). Finally, intracellular microelectrode recordings demonstrated accompanying reductions in maximum AP upstroke rates, (d*V*/d*t*)_max_, and prolonged AP conduction times in ventricles of *Pgc‐1β* ^−/−^ hearts (Ahmad et al., 2017, Ahmad, Valli, Chadda et al., 2018).

Altered (d*V*/d*t*)_max_ correlates with changes in peak Na^+^ currents (*I*_Na_), driving the AP depolarisation and the consequent conduction velocity in excitable cells (Fraser, Huang, & Pedersen, 2011; Sheikh et al., 2001; Usher‐Smith, Xu, Fraser, & Huang, 2006). Previous studies have also demonstrated that Na^+^ current can be compromised under conditions of increased reactive oxygen species (ROS) production and reductions in cytosolic NAD^+^/NADH (Liu et al., 2009; Liu, Liu, & Dudley, 2010), both conditions associated with energetic compromise (Faivre & Findlay, 1990; Fosset, De Weille, Green, Schmid‐Antomarchi, & Lazdunski, 1988; Manning, Coltart, & Hearse, 1984). Na^+^ current, AP conduction and (d*V*/d*t*)_max_ were similarly reduced with the abnormal Ca^2+^ homeostasis in proarrhythmic murine, *RyR2*‐P2328S/P2328S, hearts (Zhang et al., 2013)_._ The latter was then attributed to reduced Nav1.5 expression (Ning et al., 2016) or function (King, Wickramarachchi et al., 2013; King, Zhang et al., 2013). *Pgc‐1β* ^−/−^ cardiomyocytes similarly show altered Ca^2+^ homeostasis manifest in abnormal diastolic Ca^2+^ transients (Gurung et al., 2011).

The present experiments were designed to explore a hypothesis implicating alterations in Na^+^ channel function in the age‐dependent ventricular proarrhythmic *Pgc‐1β* ^−/−^ phenotype. Application of the loose patch technique permitted a comparison of Na^+^ against K^+^ currents in ventricular preparations from young and aged, WT, and *Pgc‐1β* ^−/−^ hearts. The loose patch‐clamp technique apposes an extracellular solution filled electrode against an intact, in situ, cardiomyocyte membrane patch without perturbing its intracellular space thereby preserving in vivo extracellular [Na^+^] and intracellular Ca^2+^ homeostatic conditions (Almers, Stanfield, & Stühmer, 1983a; King, Wickramarachchi et al., 2013; Stühmer, Roberts, & Almers, 1983). This contrasts with the cardiomyocyte isolation and intracellular Ca^2+^ chelation entailed in conventional whole‐cell patch clamp (Gurung et al., 2011; Lei et al., 2005; Martin et al., 2012). Previous loose patch clamp experiments reversibly manipulating extracellular (Na^+^) had identified early inward currents elicited by depolarising steps with Na^+^ currents determining the maximum cardiac AP upstroke rate (d*V*/d*t*)_max_, (King, Wickramarachchi et al., 2013). The present experiments could thus assess and quantify the activation, inactivation, and recovery from inactivation of inward Na^+^ currents attributable to Nav1.5. They then compared these to the corresponding activation and rectification properties of voltage‐dependent K^+^ currents in the same preparations.

## 2 MATERIALS AND METHODS

### 2.1 Animals and ethical approval

This study has been regulated under the Animals (Scientific Procedures) Act 1986 Amendment Regulations 2012 following ethical review by the University of Cambridge Animal Welfare and Ethical Review Body (AWERB). C57/B6 mice maintained in an animal facility under 12‐hr light–dark cycles at a stable temperature (21°C) were fed sterile chow (RM3 Maintenance Diet; SDS, Witham, United Kingdom) with free access to water, bedding and environmental stimuli. *Pgc‐1β* ^−/−^ mice were generated using a triple LoxP targeting vector as previously described (Lelliot et al., 2006). The four experimental groups consisted of young WT (12–16 weeks), young *Pgc‐1β* ^−/−^ (12–16 weeks), aged WT (>52 weeks) and aged *Pgc‐1β* ^−/−^ (>52 weeks) with equal numbers of patches from male and female animals studied. Before killing by cervical dislocation (Schedule 1; Animals (Scientific Procedures) Act 1986), mice were administered 200 IU of unfractionated heparin (Sigma‐Aldrich, Poole, UK) intraperitoneally. There were no recovery, anaesthetic or surgical procedures. Chemical agents were obtained from Sigma‐Aldrich unless otherwise stated.

### 2.2 Ventricular preparations

Immediately after killing, hearts were excised and placed in ice‐cold Krebs–Henseleit (KH) solution (mM): NaCl, 108; NaHCO_3_, 25; KCl, 4.0; KH_2_PO_4,_ 1.2; MgCl_2,_ 1.0; CaCl_2,_ 1.8; glucose, 10; and Na‐pyruvate, 2.0; pH adjusted to 7.4 and bubbled with 95% O_2_/5% CO_2_ (British Oxygen Company, Manchester, UK). The aorta was cannulated with a trimmed and filed 21‐G hypodermic needle, and then secured onto the perfusion cannula of a Langendorff perfusion system by an aneurysm clip and a 5‐0 braided silk suture. Retrograde perfusion under constant flow (2.1 ml/min; Watson‐Marlow, Falmouth, UK, peristaltic pump) perfused 75 ml of a KH solution to which 10 mM 2,3‐butanedione monoxime (BDM) and 10 μM blebbistatin (Cayman Chemical Company, Ann Arbor, MI) were added to give a KH‐BDM/blebbistatin solution to achieve electromechanical uncoupling. The heart was then immediately placed in ice‐cold KH‐BDM/blebbistatin solution for dissection of the right ventricle from the rest of the heart. The right ventricle was then mounted on Sylgard (Dow Chemical Company, Staines, UK) and placed in a temperature monitored experimental chamber filled with filtered KH buffer solution at 27°C.

### 2.3 Loose patch clamp experiments

Pipettes, pulled from borosilicate glass capillaries (GC150‐10; Harvard Apparatus, Cambridge, UK) using a Brown–Flaming microelectrode puller (Model P‐97; Sutter Instrument Co. Novato, CA) were mounted under a microscope with ×250 magnification and a calibrated eyepiece graticule for fracturing using a diamond knife. This applied a transverse force to the distal tip of the pipette, giving a fracture perpendicular to the pipette shaft. Selected pipettes were then fire‐polished under visual guidance at ×400 magnification using an electrically heated nichrome filament. Finally the pipette tips were bent so they made a ~45° angle with the pipette shaft. When mounted at an angle on the recording amplifier headstage they could then be made to approach the membrane vertically. Maximum internal pipette tip diameters, measured at ×1,000 magnification, were 28–32 µm following polishing.

**Figure 1:**
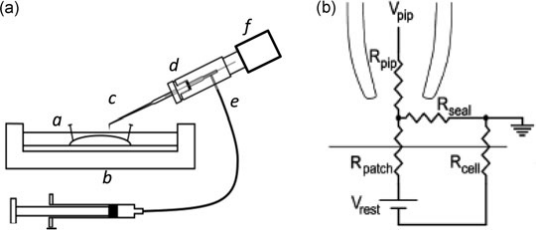
Loose patch clamping of murine ventricular myocytes. (a) Experimental loose patch configuration: (*a*) mounted muscle preparation under Krebs–Henseleit solution within (*b*) experimental chamber. (*c*) Loose patch pipette mounted at 45° to the preparation but bent to permit right‐angled contact of pipette tip with the myocyte surface. Pipette held within (*d*) half‐cell chamber in turn connected to (*e*) air line and suction syringe, and mounted on (*f*) headstage moved under micromanipulator and upright microscope guidance. (b) Equivalent circuit of loose patch clamp electrode on membrane. Pipette clamped at voltage *V*_pip_. Compensation for the voltage error arising from currents flowing through the series combination of the pipette resistance (*R*_pip_) and the seal resistance (*R*_seal_) achieved using a bridge circuit in the custom‐designed loose patch clamp amplifier. As the loose patch technique alters the extracellular potential within the patch relative to RMP, negative and positive voltage excursions in *V*_pip_, respectively, produce hyperpolarising and depolarising voltage steps relative to RMP. RMP: resting membrane potential; *R_patch_*: patch membrane resistance; *R_cell_*: overall cell membrane resistance

The distal ends of the pipette were filled with KH solution, and then mounted onto a pipette holder incorporating an Ag/AgCl half cell connected to the headstage. This lowered the pipette onto the membrane surface. Gentle suction was then applied through an air‐filled line connecting the pipette holder and a syringe. This resulted in seal formation with a membrane patch. Figure 1 illustrates (a) the recording layout and (b) the equivalent circuit resulting from apposing the loose patch clamp electrode to the membrane surface. Voltage‐clamp steps were applied under computer control. Owing to its relatively low seal resistances, the loose patch clamp results in larger leakage currents compared with those associated with conventional patch clamping. Most of the leakage current, series resistance errors and displacement currents through the pipette capacitance were compensated for using a custom‐built amplifier (Stühmer et al., 1983). Residual leakage and capacitive currents were then corrected by using reference records obtained from subsequent P/4 control protocols that applied voltage steps with amplitudes scaled down by a factor of 4 in a direction opposite to those of the test steps as previously described (Almers et al., 1983a; Almers, Stanfield, & Stühmer, 1983b). Each established patch was then subject to a complete set of pulse procedures examining either inward or outward current activation, which could be accomplished within 30 s making effects arising from prolonged changes in the patch such as bleb formation unlikely (Milton & Caldwell, 1990).

The loose patch clamp controls the voltage at the extracellular surface of the membrane within the seal in an intact cardiomyocyte. Accordingly, positive and negative voltage steps applied through the pipette respectively hyperpolarise and depolarise the membrane potential relative to the cardiomyocyte resting membrane potential (RMP). Voltage steps are therefore described in terms of the alterations they produce relative to the RMP, following the convention in earlier studies that introduced this technique (Almers et al., 1983a, 1983b). Construction of current–voltage curves used values of current densities (pA/μm^2^) obtained by normalizing the observed currents (nA) to the cross sectional area (*πa* ^2^) of the pipette tip, radius *a*. Values in inactivation curves plotted observed maximum currents normalized to the maximum currents obtained at the most polarised holding potentials. Curve‐fitting procedures of both plots against membrane potential used the open source fitting algorithm in the open source R programming language.

Statistical analysis of results applied two‐way analysis of variance (ANOVA) to the experimental groups of young and aged, WT and *Pgc‐1β* ^−/−^ preparations to test for significant differences arising from independent or interacting effects of age and/or genotype on fitted parameters. The presence of such differences was then explored by pairwise tests for differences using Tukey’s honestly significant difference testing.

## 3 RESULTS

### 3.1 Currents reflecting ventricular Na^**+**^ channel activation

**Figure 2:**
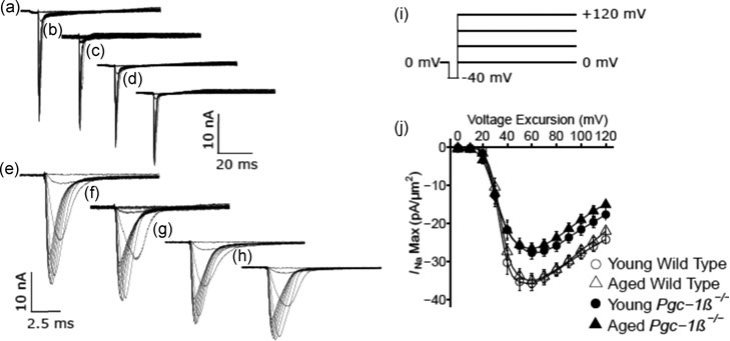
Activation properties of voltage‐dependent inward Na^+^ currents of murine ventricular preparations under loose patch clamp. Typical records are shown for young (a,c,e,g) and aged (b,d,f,h) wild‐type (a,b,e,f),and *Pgc‐1β* ^−/−^ hearts (c,d,g,h),at slow (a–d) and fast (e–g) time bases in response to (i) activation pulse protocols beginning from the RMP. This first applied a 5 ms duration prepulse 5 ms into the recording period to (RMP − 40 mV). It then applied successively larger depolarising test voltage steps increased in +10 mV increments up to (RMP + 120 mV). (j) Peak currents, *I*_NaMax,_ are plotted against the voltage excursion for young (circles) and aged (triangles), wild‐type (clear symbols) and *Pgc‐1β* ^−/−^ ventricular myocytes (filled symbols). RMP: resting membrane potential

Isolated ventricular preparations were used for loose patch‐clamp recordings. Typical results of Na^+^ current activation are shown in Figure 2. Comparisons were made between experimental groups that consisted of preparations from young (panels a,c,e,g) and aged (b,d,f,h), wild‐type (WT; a,b,e,f) and *Pgc‐1β* ^−/−^ hearts (c,d,g,h). Displays of the resulting current traces are shown at slow (a–d) timebases illustrating the full time course of the current records. Those at rapid (e–h) timebases show the detailed kinetics of the transient components of the observed currents. The pulse protocol (Figure 2i) used first held the patches at the RMP for 5 ms from the beginning of the 80 ms recording period. A 5 ms prepulse step to *V*_0_ = (RMP −40 mV) was then applied to remove any residual Na^+^ current inactivation and ensure a consistent initial state of activation of Na^+^ channels within the patch. This was then followed by the depolarising test steps that extended to the end of the 80 ms record length; these were of successively larger amplitudes through the 13 successive sweeps that were applied, and were made to voltages ranging from *V*_1_ = RMP to *V*_1_ = (RMP + 120 mV), in successive +10 mV increments. The observed currents were corrected for residual leakage by the P/4 protocol to give a family of traces reflecting the voltage dependence of Na^+^ channel activation, with inward currents shown as downward, negative deflections.

The resulting current traces typically began with a small consistent upward deflection in response to the −40 mV prepulse (a–d). The inward currents obtained in response to the subsequent test voltage steps to level *V*_1_ illustrate the typical Na^+^ current activation characteristics expected. The recorded currents initially increased with time to a peak value that became greater with greater voltage excursion and varied nonlinearly with voltage *V*_1_. Each peak was then followed by an inactivation decay whose characteristics were similarly determined by the voltage *V*_1_ (e–h). The records demonstrated that ventricular preparations from young and aged mice showed similar Na^+^ current amplitudes. However, *Pgc‐1β* ^−/−^ ventricular preparations showed consistently smaller Na^+^ currents than did WT preparations.

### 3.2 Currents reflecting ventricular Na^**+**^ channel inactivation

**Figure 3:**
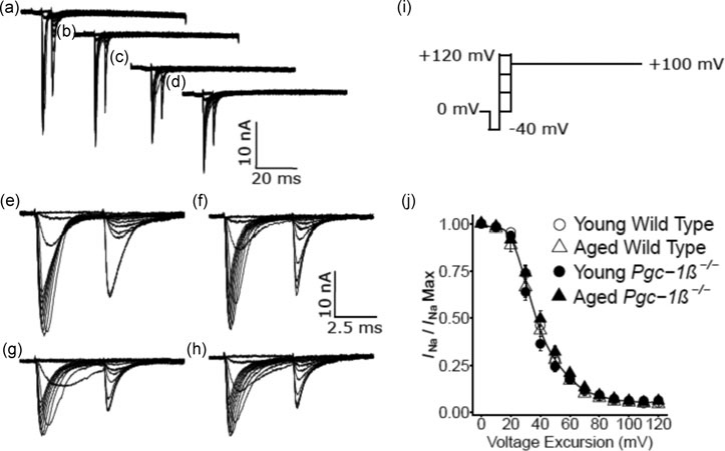
Inactivation properties shown by voltage‐dependent inward Na^+^ currents under loose patch clamp of murine ventricular preparations. Typical records shown for young (a,c,e,g) and aged (b,d,f,h) wild‐type (a,b,e,f), and *Pgc‐1β* ^−/−^ ventricular preparations (c,d,g,h), at slow (a–d) and fast (e–g) time bases in response to inactivation pulse protocols. (i) In the pulse protocol, a prepulse (duration 5 ms) was applied from the RMP, 5 ms into the recording period to (RMP − 40 mV). This was followed by successively larger depolarising 5 ms duration conditioning voltage steps increased in +10 mV increments up to (RMP + 120 mV). Finally, in all sweeps, the voltage finally was stepped to a constant test level of (RMP + 100 mV). The resulting peaks of the Na^+^ currents were then quantified to characterise the inactivation brought about by the preceding conditioning step. (j) Peak currents *I*_NaMax_ plotted against voltage excursion for the conditioning voltage step in young (circles) and aged (triangles), wild‐type (clear symbols), and *Pgc‐1β* ^−/−^ ventricular preparations (filled symbols). RMP: resting membrane potential

Results from experiments exploring ventricular Na^+^ current inactivation properties are illustrated in Figure 3. Again, the experimental groups consisted of preparations from young (panels a,c,e,g),and aged (b,d,f,h), WT (a,b,e,f), and *Pgc‐1β* ^−/−^ mice (c,d,g,h). In the pulse protocols (Figure 3i), patches were first held at the RMP for 5 ms. This was followed by a prepulse of 5‐ms duration to *V*_0_ = (RMP − 40 mV). Next, depolarising conditioning steps were applied to voltages that were altered in 10 mV increments between *V*_1_ = RMP to (RMP + 120 mV) through the 13 successive sweeps. This conditioning step elicited a Na^+^ current reflecting the process of activation followed by inactivation, with the extent of the latter determined by the voltage *V*_1_. The extent of inactivation was assessed by imposition of the test step 5 ms later to a fixed voltage *V*_2_ = (RMP + 100 mV). This voltage was maintained to the end of the record length. The resulting traces (Figure 3a–h) reflect Na^+^ currents which would become reduced in amplitude to an extent dependent upon the channel *inactivation* brought about by the prior voltage prepulse to *V*_1_. This combined pulse protocol thus gave families of currents in response to the step to voltage *V*_2_, the peak currents of which vary nonlinearly and decrease in amplitude as voltage *V*_1_ is varied. These also demonstrated that the magnitudes of the observed currents in preparations from young and aged mice were similar, but currents were consistently reduced in *Pgc‐1β* ^−/−^ relative to WT ventricular preparations.

### 3.3 Voltage dependence of ventricular Na^**+**^ current activation

Figure 2j plots current–voltage curves quantifying the activation of peak ventricular Na^+^ current (mean ± *SEM*) with excursion to the test voltage *V*_1_. It compares results from preparations from young (circles) and aged (triangles), WT (open symbols), and *Pgc‐1β* ^−/−^ mice (filled symbols). In such activation plots, the peak inward Na^+^ current, *I*_Na_, increased with the amplitudes of the depolarising steps beyond +10 mV, reaching a maximum value at a voltage excursion at around +80 mV. Their amplitudes then decreased with larger voltage excursions as these reached voltages *V*_1_ successively closer to the Na^+^ Nernst potential.

The activation of such peak Na^+^ currents, *I*, was empirically related to the activating voltage *V* = *V*_1_ by a Boltzmann function expressed in terms of the maximum value of the peak current, *I*_max_, voltage at half maximal peak current, V*, and a steepness factor describing the voltage sensitivity of the current, *k*: *I* = *I*_max_{1 − 1/{1 + exp [(*V* − V*)/*k*]}}. The maximum values of the peak ventricular inward Na^+^ currents were similar in young WT (−36.63 ± 2.14 [*n* = 20] pA/μm^2^) and aged WT (− 35.43 ± 1.96 [*n* = 18] pA/μm^2^) mice. They were reduced to similar extents in young (− 29.06 ± 1.65 [*n* = 23] pA/μm^2^) and aged *Pgc‐1β* ^−/−^ mice (− 27.93 ± 1.63 [*n* = 20] pA/μm^2^). These changes reflected independent effects of genotype (*F* = 16.57; *p* = 0.0001), but not of age (*F* = 0.40; *p* = 0.53), or of interacting effects of age and genotype (*F* < 0.001; *p* = 0.99) on two‐way ANOVA. Post hoc Tukey’s tests demonstrated pairwise differences between aged WT and aged *Pgc‐1β* ^−/−^ hearts (*p* = 0.033), young WT and young *Pgc‐1β* ^−/−^ hearts (*p* = 0.019), as well as young WT and aged *Pgc‐1β* ^−/−^ hearts (*p* = 0.0075).

In contrast, V* values were similar amongst ventricular preparations from young (34.28 ± 1.10 [*n* = 20] mV) and aged WT (34.49 ± 0.85 [*n* = 18] mV), and young (34.49 ± 1.80 [*n* = 23] mV) and aged *Pgc‐1β* ^−/−^ hearts (32.22 ± 1.87 [*n* = 20] mV). Accordingly, two‐way ANOVA demonstrated that there were no independent effects of either genotype (*F* = 0.37; *p* = 0.54) or age (*F* = 0.52; *p* = 0.47). Nor were there interacting effects of age and genotype (*F* = 0.65; *p* = 0.42). Values of *k* were also similar amongst ventricular preparations from young (3.24 ± 0.25 [*n* = 20] mV) and aged WT (4.06 ± 0.18 [*n* = 18] mV), and young (4.04 ± 0.28 [*n* = 23] mV) and aged *Pgc‐1β* ^−/−^ hearts (4.11 ± 0.24 [*n* = 20] mV). Two‐way ANOVA indicated no independent effects of either genotype (*F* = 3.23; *p* = 0.072) or age (*F* = 2.96; *p* = 0.089). Nor were there interacting effects of age and genotype (*F* = 2.34; *p* = 0.13).

### 3.4 Voltage dependence of ventricular Na^**+**^ current inactivation

Figure 3j plots the voltage dependences of Na^+^ current inactivation of preparations from young (circles) and aged (triangles), WT (open symbols) and *Pgc‐1β* ^−/−^ mice (filled symbols). The peak inward Na^+^ currents observed in response to a depolarising step to a constant test voltage decreased as the preceding prepulse voltages, *V*_1_, became progressively positive. This reflects an inactivation process increasing in extent with progressive depolarisation. The peak currents were related to an inactivation function in the inactivating voltage *V* = *V*_1_ through a Boltzmann function of the form: *I* = *I*_max_ /{1 + exp[(*V* − V*)/*k*]}.

Values of V* were similar amongst young (39.33 ± 1.32 [*n* = 20] mV) and aged WT (37.41 ± 2.20 [*n* = 18] mV), and young (36.10 ± 1.26 [*n* = 23] mV) and aged *Pgc‐1β* ^−/−^ preparations (40.39 ± 1.72 [*n* = 20] mV) with an absence of independent effects of either genotype (*F* = 0.040; *p* = 0.84) or age (*F* = 0.714; *p* = 0.401), or interacting effects of age and genotype (*F* = 3.66; *p* = 0.059) on two‐way ANOVA. Values of *k* were also similar amongst young (8.11 ± 0.53 [*n* = 20] mV) and aged WT (7.48 ± 0.68 [*n* = 18] mV), and young (7.85 ± 0.58 [*n* = 23] mV) and aged *Pgc‐1β* ^−/−^ preparations (9.36 ± 0.42 [*n* = 20] mV), with an absence of independent effects of either genotype (*F* = 1.75; *p* = 0.19) or age (*F* = 0.81; *p* = 0.37), or interacting effects of age and genotype (*F* = 3.66; *p* = 0.060).

Thus, in contrast to the genotype dependent effect upon the maximum peak Na^+^ currents *I*_max_, neither age or genotype influenced the voltages at half maximal current V*, or the steepness factors, *k*, of Boltzmann functions describing current activation or current inactivation.

### 3.5 Time course of Na^**+**^ channel recovery from inactivation

**Figure 4:**
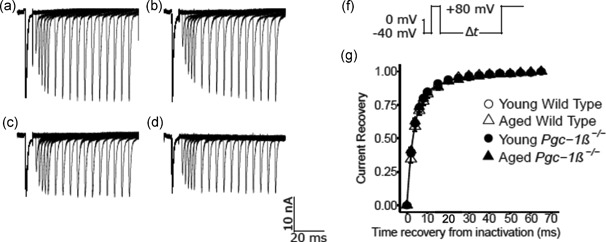
Currents illustrating Na^+^ channel recovery from inactivation following restoration of the membrane potential. Records are shown for young (a,c) and aged (b,d) wild‐type (a,b) and *Pgc‐1β* ^−/−^ ventricular preparations (c,d). In the pulse protocols (f) the membrane voltage was first held at the RMP for 1 ms from the beginning of the recording period. A hyperpolarising prepulse was then imposed to (RMP − 40 mV). This was then followed by a 5 ms duration P1 conditioning step to (RMP + 80 mV). The subsequent 5 ms duration test steps to (RMP + 80 mV) that followed were imposed after different time intervals, ∆*T*, between 5 ms and 65 ms in 5 ms increments through the 12 successive sweeps making up the protocol. (g) Plots of the recovery of peak *I*_Na_ against time intervening between termination of the conditioning and imposition of the test pulse

Figure 4 shows typical experimental results of the time course of recovery from inactivation. This was explored by restoration of the baseline voltage after an initial conditioning depolarising step to a fixed voltage. Results are compared for preparations from young (a,c) and aged (b,c),WT (a,b) and *Pgc‐1β* ^−/−^ hearts (c,d). The pulse protocol (Figure 4f) held the membrane potential at the RMP for 1 ms from the beginning of the recording period. This was followed by a hyperpolarising prepulse to voltage *V*_0_ = (RMP − 40 mV) for 4 ms to establish consistent baseline levels of Na^+^ current inactivation as in the previous protocols.

A 5 ms P1 conditioning step was then imposed between *V*_0_ and *V*_1_ = (RMP + 80 mV). This elicited Na^+^ current activation followed by its inactivation decay, before restoration of the baseline voltage *V*_0_. The depolarising 5 ms duration P2 steps to voltage *V*_3_ = (RMP + 80) mV were then imposed after different time intervals, ∆T. This time interval varied between 2 and 75 ms, altered in 2 ms increments for the first five recordings and 5 ms increments thereafter, through the 16 successive sweeps making up the protocol. These P2 steps elicited a Na^+^ current activation whose peak amplitude, normalised to corresponding values in the P1 step, reflected the Na^+^ current recovery from inactivation with time ∆*T*. Time constants, *τ*, were fitted to the exponential function *I* = *I*_max_(1 – exp(−∆*T*/*τ*)) describing this recovery (Figure 4g). Values of *τ* were similar amongst young (4.69 ± 0.47 [*n* = 20] ms) and aged WT (5.31 ± 0.58 [*n* = 18] ms), and young (4.58 ± 0.27 [*n* = 23] ms) and aged *Pgc‐1β* ^−/−^ hearts (4.45 ± 0.29 [*n* = 20] ms). There was an absence of any effects of genotype (*F* = 1.31; *p* = 0.26), age (*F* = 0.30; *p* = 0.59), or interacting effects of age and genotype (*F* = 0.88; *p* = 0.35) on *τ* with two‐way ANOVA.

### 3.6 Voltage dependences of ventricular K^**+**^ current activation

The loose patch‐clamp technique was used to investigate voltage‐dependent total outward, K^+^, currents and their rectification properties in murine ventricular preparations for the first time. They demonstrated that, in contrast to the differences in maximum Na^+^ current, these outward current properties were similar amongst groups.

**Figure 5:**
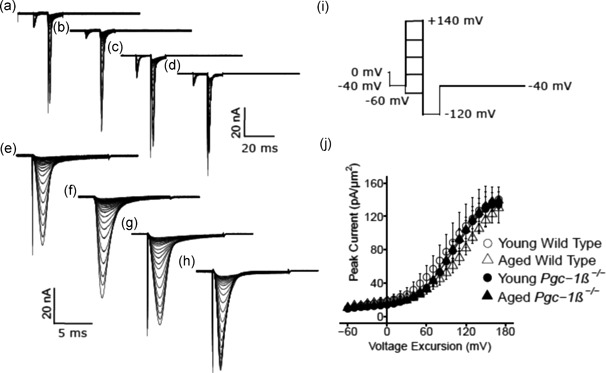
Activation properties of K^+^ currents as reflected in tail currents in ventricular preparations. This shows records from young (a,c,e,g) and aged (b,d,f,h) WT (a,b,e,f) and *Pgc‐1β* ^−/−^ ventricular preparations (c,d,g,h), at slow (a–d) and fast (e–g) time bases. The pulse procedure (i) first applied a voltage step from RMP to (RMP – 20 mV) between 1 and 10 ms following commencement of the recording period. This was followed by 10 ms duration test steps to voltages between (RMP – 60 mV) and (RMP + 140 mV) incremented in 10 mV steps through the 21 sweeps investigated. A final 10 ms duration hyperpolarising step to (RMP – 120 mV) was then imposed. This gave tail currents reflecting the preceding K^+^ current activation. Finally, the membrane potential was restored to (RMP − 20 mV). (j) Plot of the maximum amplitude of the tail currents against voltage excursion in the young (circles) and aged (triangles) WT (open symbols) and *Pgc‐1β* ^−/−^ ventricular preparations (filled symbols). WT: wild‐type

Figure 5 illustrates the results of experiments investigating K^+^ current activation in preparations from young (Figure 5a,c,e,g) and aged (Figure 5b,d,f,h), WT (a,b,e,f), and *Pgc‐1β* ^−/−^ (c,d,g,h) mice. The pulse procedure (Figure 5i) initially imposed a voltage step between 1 and 10 ms from the start of the recording period from RMP to (RMP – 20 mV). It then imposed a 10 ms duration test step to voltages between (RMP – 60 mV) and (RMP + 170 mV), varied in increments of 10 mV through the 24 sweeps that were investigated. The traces shown at slow (a–d) sweep speeds encompassing the entire record demonstrate that such test steps initially elicited Na^+^ channel activation, followed by its inactivation. This was succeeded in some traces by a gradual outward current reflecting activation of a rectified voltage‐dependent K^+^ current. This was followed by a 10 ms hyperpolarising step to a fixed voltage of (RMP – 120 mV), before final restoration of the membrane potential to (RMP − 20 mV). The traces shown at the rapid timebases (e–h) demonstrate that this resulted in K^+^ tail currents. This reflects the instantaneous conductance resulting from K^+^ channel activation induced by and existing at the end of the preceding depolarising step. The amplitudes of these families of tail currents were similar between experimental groups.

Figure 5j plots typical activation K^+^ current‐voltage curves for the young (circles) and aged (triangles) WT (open symbols) and *Pgc‐1β* ^−/−^ ventricular preparations (filled symbols). The plots were close to superimposable, their areas with the abscissa showing neither independent (*F* = 0.02; *p* = 0.89 and *F* = 0.19; *p* = 0.67, respectively) nor interacting (*F* = 0.42; *p* = 0.52) effects of genotype or age.

### 3.7 Rectification properties of ventricular K^**+**^ currents

**Figure 6:**
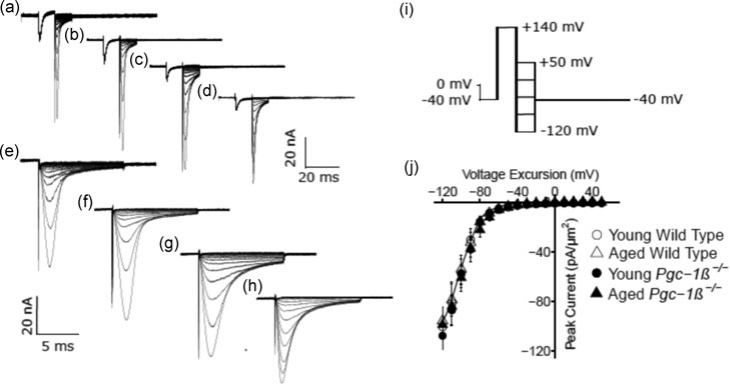
K^+^ current rectification properties reflected in tail currents in ventricular preparations. Typical records shown from young (a,c,e,g) and aged (b,d,f,h) WT (a,b,e,f) and *Pgc‐1β* ^−/−^ ventricles (c,d,g,h), at slow (a–d) and fast (e–g) time bases. The pulse procedure (i) first applied a voltage step from RMP to (RMP – 20 mV) between 1 and 10 ms after the beginning of the recording period. This was followed by a 10 ms duration test step to a fixed voltage of (RMP + 140 mV). This was succeeded by a 10 ms duration step to varying voltages between (RMP − 120 mV) and (RMP + 50 mV). The latter resulted in tail currents which could be plotted against voltage to obtain (j) instantaneous current–voltage relationships reflecting the rectification properties of the activated channel in the young (circles) and aged (triangles) WT (open symbols) and *Pgc‐1β* ^−/−^ ventricles (filled symbols). Finally, the membrane potential was restored to (RMP − 20 mV). WT: wild‐type

Figure 6 illustrates results from investigations of K^+^ current rectification properties in ventricular preparations from young (Figure 6a,c,e,g) and aged (Figure 6b,d,f,h), WT (a,b,e,f), and *Pgc‐1β* ^−/−^ (c,d,g,h) hearts. The pulse procedure (Figure 6i) initially imposed a voltage step between 1 and 10 ms from the start of the recording period from RMP to (RMP – 20 mV). It then imposed a 10 ms test step to a fixed voltage of (RMP + 140 mV). As indicated in the traces displayed at a compressed timebase illustrating the entire record (a–d), such test steps initially elicited an inward, Na^+^ current, activation, followed by its inactivation, and this was followed by a specific level of a rectified K^+^ current activation. Further test steps to a range of voltages between (RMP − 120 mV) and (RMP + 50 mV) altered in 10 mV increments through successive sweeps then elicited tail currents, shown at low (a–d) and high sweep speeds (e–h), that reflect the instantaneous current–voltage relationship and the rectification properties of the activated channel (Figure 6j). Plots of such currents against voltage (Figure 6j) showed the typical concave downward form of K^+^ channel rectification. These were similar amongst the young (circles) and aged (triangles) WT (open symbols) and *Pgc‐1β* ^−/−^ ventricular preparations (filled symbols), demonstrating close to superimposable plots and enclosing areas with the abscissa in which there were neither independent (*F* = 0.18; *p* = 0.67 and *F* = 0.004; *p* = 0.95, respectively) nor interacting (*F* = 0.013; *p* = 0.91) effects of genotype or age.

## 4 DISCUSSION

Ventricular arrhythmic risk is known to increase with age (Adabag et al., 2015; Hookana et al., 2011; Kucharska‐Newton et al., 2010) through accumulation of mitochondrial genomic mutations and impaired autophagy (Michikawa, Mazzucchelli, Bresolin, Scarlato, & Attardi, 1999; Pyo, Yoo, & Jung, 2013). It also increases with clinically common age‐related metabolic conditions arising from obesity, metabolic syndrome, diabetes mellitus, and heart failure, which constitute independent risk factors for such outcomes. Biochemical effects of the latter conditions were previously replicated in murine *Pgc‐1β* ^−/−^ hearts which demonstrated deficiencies in key mitochondrial energetic tricarboxylic acid cycle, fatty acid β‐oxidative, and oxidative phosphorylative processes (Arany et al., 2005; Finck & Kelly, 2006; Lin et al., 2005).

More recent reports on *Pgc‐1β* ^−/−^ hearts have also demonstrated age‐dependent ventricular proarrhythmic phenotypes (Gurung et al., 2011) accompanying slowed propagation of the AP mediating cardiomyocyte excitation (Ahmad, Valli, Salvage et al., 2018). This was accompanied by compromised maximum AP depolarization rates, (d*V*/d*t*)_max_ and an age‐dependent accentuation of fibrotic change accentuated by the *Pgc‐1β* ^−/−^ genotype (Ahmad et al., 2017, Ahmad, Valli, Chadda et al., 2018). Proarrhythmic reductions in conduction velocity have been attributed to compromised cardiac Na^+^ channel function in genetically modified *Scn5a* ^*+/−*^ murine models modelling the Brugada Syndrome (Huang, 2017; Kalin, Usher‐Smith, Jones, Huang, & Sabir, 2010; Martin, Grace, & Huang, 2011; Martin, Guzadhur, Grace, Lei, & Huang, 2011; Sabir, Killeen, Grace, & Huang, 2008). They may also arise from fibrotic change altering cardiomyocyte capacitance following cardiomyocyte‐fibroblast fusion (Davies et al., 2014; Mahoney, Mezzano, & Morley, 2016; Xie et al., 2009), or increased intercellular gap junction resistance with connexin Cx40 or Cx43 deficiency accompanying or resulting from tissue fibrosis (Jeevaratnam et al., 2011, 2012).

The present experiments accordingly test a hypothesis implicating compromised Na^+^ channel function as a contributor to such arrhythmic substrate. Previous reports had suggested that metabolic stress of the kind occurring with the *Pgc‐1β* ^−/−^ genotype could potentially alter Na^+^ channel activity through increased ROS production (Liu et al., 2010), or compromised NAD^+^–NADH ratios (Pugh et al., 2013), effects which are reversed by the mitochondrial ROS scavenger mitoTEMPO (Liu, Liu, & Dudley, 2010) and NAD^+^ restoration (Gomes et al., 2013), respectively. *Pgc‐1β* ^*−/−*^ cardiomyocytes also show altered Ca^2+^ homeostasis (Gurung et al., 2011) accompanied by increased frequency of proarrhythmic delayed after depolarisation activity in common with murine *RyR2*‐P2328S ventricular cardiomyocytes (Goddard et al., 2008).

However, *RyR2*‐P2328S ventricles also showed parallel reductions in AP conduction velocity and maximum depolarization rates (Zhang et al., 2013). These were subsequently correlated with reduced Na^+^ current (King, Wickramarachchi et al., 2013), itself attributed to both chronically downregulated Nav1.5 expression (King, Wickramarachchi et al., 2013; Ning et al., 2016) and acute (King, Wickramarachchi et al., 2013; King, Zhang et al., 2013; Zhang et al., 2013) but potentially reversible loss of Nav1.5 function (Knollmann et al., 2001; Salvage et al., 2015, 2018). Previous studies have reported that increasing or sequestrating intracellular (Ca^2+^) reduced or restored Na^+^ currents and (d*V*/d*t*)_max_, respectively, in in vitro WT cardiomyocytes (Casini et al., 2009). Altered cellular Ca^2+^ homeostasis could potentially acutely affect Nav1.5 function (Aiba et al., 2010; Ashpole et al., 2012; Tan et al., 2002) through its C‐terminal region, either directly at an EF hand motif (Wingo et al., 2004) or indirectly through an IQ domain sensitive to calmodulin/calmodulin kinase II (CaMKII; Mori et al., 2000). Nav1.5 also shows multiple phosphorylatable, serine 516 and 571, and threonine 594 sites in its DI‐II linker that are targetable by CaMKII action (Grandi & Herren, 2014; Mori et al., 2000; Wagner et al., 2011).

The loose patch‐clamp method measures transmembrane current flow into an extracellular electrode apposed to the cardiomyocyte surface membrane forming the patch. It further could be applied to intact ventricular tissue preparations (King, Wickramarachchi et al., 2013; Ning et al., 2016; Salvage et al., 2015). It thereby avoided the tissue disruption, cell isolation, and intracellular Ca^2+^ chelation required by conventional whole‐cell patch‐clamp studies (Lei et al., 2005; Martin et al., 2012). The latter may account for differences between some of the present findings and previous observations using conventional patch clamping of isolated cells (Gurung et al., 2011). The present studies further used in vivo rather than reduced extracellular [Na^+^] levels thereby sparing effects on Na^+^–Ca^2+^ exchange. Previous reports had identified early inward currents obtained in loose patches with Na^+^ currents mediating AP upstroke and its conduction (King, Wickramarachchi et al., 2013).

Depolarising test steps elicited inward currents which activated to a peak value then showed an inactivating decline to baseline in a pattern characteristic of voltage‐dependent Na^+^ currents. These features were observed in all the young and aged, WT and *Pgc‐1β* ^−/−^ ventricular cardiomyocytes studied. The resulting current–voltage relationships gave values that increased with depolarisation up to voltage excursions of (RMP + 80 mV) beyond which they declined as expected toward the Na^+^ reversal potential. In contrast, imposition of voltage steps exploring inactivation characteristics began with prepulse voltages to varying levels of depolarisation which similarly elicited currents rising to a peak followed by an inactivation‐mediated decay. These were followed by steps to a fixed depolarised membrane potential. The latter elicited currents whose peaks declined in amplitude with the depolarising prepulse as expected from the voltage dependent inactivation this would produce. Inactivation curves were constructed from plotting such peak currents against the prepulse level. These gave currents that fell with depolarisation. A two‐way ANOVA, followed where indicated by post‐hoc pairwise testing, then examined for significant independent and interacting effects of the *Pgc‐1β* ^−/−^ (as opposed to WT) genotype and of age on the quantitative parameters emerging from fitting Boltzmann relationships to both the activation and inactivation data. This demonstrated independent effects of genotype, but not of age, or of interactions between these factors, in reducing maximum values of the peak Na^+^ currents. However, there were neither independent nor interacting effects on consequently similar values of voltage at half maximum current, V*, and the steepness *k* of both activation and inactivation characteristics. Nor were there differences in the time constants describing the timecourses for recovery from inactivation following restoration of the background voltage, which were consequently similar between experimental groups. These findings suggested that the altered availability of functional Na^+^ channels accompanied otherwise normal voltage dependences.

These differences specifically in maximum Na^+^ current contrasted with indistinguishable outward K^+^ current characteristics between groups. Pulse protocols investigating the voltage dependences of K^+^ current activation applied voltage steps to a range of test potentials, and followed these with hyperpolarising steps that would elicit a tail current whose amplitude reflected the preceding K^+^ channel activation. Conversely K^+^ current rectification properties were investigated by first imposing a conditioning step to a fixed membrane voltage step to produce a constant level of activation. This was followed by voltage steps to varying membrane potentials. The latter would result in tail currents flowing through similarly activated channels, in response to different driving forces, which would vary with the voltage dependence of the resulting open channel rectification properties. Both experiments yielded similar ventricular cardiomyocyte currents suggesting similar activation and rectification characteristics in all the four experimental groups explored. These thus yielded closely concordant activation and instantaneous current–voltage curves. Thus, two‐way ANOVA demonstrated that there were neither independent nor interacting effects of genotype or age on areas made between observed values representing either plots of K^+^ current activation, or of rectification properties and their voltage abscissae.

The present current–voltage analyses of both activation and inactivation properties thus associate the *Pgc‐1β^−/−^* genotype, but not ageing, with alterations in maximum Na^+^ conductance. This took place in an absence of alterations in Na^+^ channel activation and inactivation properties reflected in their voltages at half maximum current, V* and the steepness *k*, of their voltage dependences, their timecourses of recovery from inactivation, and in the voltage dependences of K^+^ current activation and rectification. The resulting reduction specifically in maximum Na^+^ current constitutes a mechanism for the observed proarrhythmic reductions in AP conduction velocity accompanied by reduced peak AP upstroke rates (d*V*/d*t*)_max_ in the presence of the *Pgc‐1β* ^−/−^ genotype (Ahmad et al., 2017).
